# The Long-Term Effects of 12-Week Intranasal Steroid Therapy on Adenoid Size, Its Mucus Coverage and Otitis Media with Effusion: A Cohort Study in Preschool Children

**DOI:** 10.3390/jcm11030507

**Published:** 2022-01-20

**Authors:** Aleksander Zwierz, Krystyna Masna, Krzysztof Domagalski, Paweł Burduk

**Affiliations:** 1 Department of Otolaryngology, Phoniatrics and Audiology, Faculty of Health Sciences, Ludwik Rydygier Collegium Medicum, Nicolaus Copernicus University, 85-168 Bydgoszcz, Poland; krymasna@gmail.com (K.M.); pburduk@wp.pl (P.B.); 2 Department of Immunology, Faculty of Biological and Veterinary Sciences, Nicolaus Copernicus University, 87-100 Torun, Poland; krydom@umk.pl

**Keywords:** adenoid hypertrophy, mucus, endoscopic evaluation, topical steroids, intranasal steroids

## Abstract

Background: The purpose of this study is to analyse the long-term effects of a 12-week course of topical steroids on adenoid size and its mucus using endoscopy and on middle ear effusion measured by tympanometry. Methods: The study presents an endoscopic choanal assessment of the change in adenoid size (adenoid to choanae ratio, A/C ratio) and its mucus coverage in 165 children with Grade II and III adenoid hypertrophy three to six months after finishing a 12-week course of intranasal steroid treatment with mometasone furoate. Additionally, tympanometry was performed to measure middle ear effusion. Changes in the tympanograms were analysed. Results: The mean A/C ratio before treatment was 65.73%. Three to six months after finishing a 12-week course of intranasal steroid treatment, the mean A/C ratio decreased to 65.52%, although the change was not statistically significant (*p* = 0.743). There was no change in adenoid mucus according to the MASNA scale before and three to six months after the end of the steroid treatment (*p* = 0.894). Long-term observations of tympanograms before and three to six months after the end of the treatment did not show improvement (*p* = 0.428). Conclusions: The results indicate that there was no effect of topical steroids on adenoid size, its mucus and otitis media with effusion (OME) three to six months after finishing a 12-week course of treatment. In the light of performed study, decision of adenoidectomy and tympanostomy should not be procrastinated.

## 1. Introduction

Nasal obstructions, recurrent upper respiratory tracts infections, mouth breathing, persistent rhinorrhoea, snoring, nasal voice and recurrent otitis media in preschool children suggest enlarged adenoids (pharyngeal tonsil) and incline pediatricians to refer the patient to an ear, nose and throat (ENT) specialist [1]. The percentage of adenoid hypertrophy in young children admitted to ENT outpatient clinics because of nasal obstructions is estimated to be 57.7% [2]. Upon confirmation of an enlarged adenoid and its symptoms, conservative treatment with the use an intranasal steroid and saline irrigation should be applied [3,4,5]. The study concerning on number of eosinophils of allergic rhinitis children demonstrate that combined steroid and saline treatment improves the efficacy of treatment [5]. Unfortunately, almost 90% of children with adenoid hypertrophy and adenoid symptoms undergo surgery in the two-year period after the initial diagnosis [6]. Adenoidectomy is also recommended for children suffered from bilateral OME lasting for over 3 months of evolution, or unilateral OME lasting for over 6 months of evolution as a surgical restoration of physiological tubal function and combined surgery of ventilation tubes installation [7]. From the other side, effectiveness of adenoid surgery in children with recurrent or chronic nasal symptoms remains unclear and may not be effective treatment in children with sinusitis [8]. In fact, adenoidectomy is one of most frequently performed surgeries in children [9]. This is particularly puzzling since numerous publications have shown the beneficial effect of conservative treatment of intranasal steroids on reducing the size of the pharyngeal tonsil and related symptoms [1,3,10,11,12,13,14]. The impact of corticosteroids on reducing adenoid tissue proliferation was also confirmed by in vitro clinical trials [15]. Two human isoforms of glucocorticoid receptors (GCR-α and GCR-β) have been identified in adenoid tissue that play a role in glucocorticoid ligand efficacy [16]. However, there are limited studies on how long the effects of intranasal steroids on adenoids persist.

In this study, we aimed to analyse changes in the size of Grade II and III pharyngeal tonsil hypertrophy its mucus coverage and middle ear effusion controlled by tympanometry three to six months after finishing a 12-week course of intranasally administered mometasone furoate.

## 2. Materials and Methods

### 2.1. Study Population

We performed retrospective analysis of data from two sequential visits of 165 preschool children aged 3–6 years (mean 4.14; SD = 0.97) before and three to six months after finishing a course of 12-week intranasal steroid therapy. We enrolled patients in the study who were classified at the first visit by fibroscopic examination as Grade 2 and 3 adenoid hypertrophy according to Bolesławska [17]. The study group consisted of 83 girls and 82 boys admitted to an ENT outpatient clinic between 2016 and 2021. We eliminated children from the study who had a history of epistaxis, immunodeficiency and hypersensitivity or allergy or atopy to mometasone furoate. Additionally, we excluded patients with genetic diseases (Down, Treacher–Collins Syndrome), craniofacial anomalies, nasal septal deviation, nasal polyp, or inferior turbinate hypertrophy. Children who had an active upper respiratory infection within two weeks of enrolling in the study or those who had previously undergone adenoidectomy or tympanostomy tube insertion were excluded from the study. We further excluded six children from the analysis because of irritation of the nose and throat, crusting, transient dryness and epistaxis, which made it necessary to discontinue the course of intranasal steroids.

### 2.2. Methods

Each child was examined twice before and after the 12-week course of steroid administration, with a period of at least three to six months without steroid intake before the visits. Because seasonality may influence adenoid mucus coverage and tympanometry, for detailed analyses patients was divided to two subgroups of thermal sequence examination, in which 85 children were first examined in winter and then in summer, and the remaining 80 children were first examined in summer and then in winter [18]. Each patient enrolled in the study underwent a parental questionnaire, history and physical examination, nasopharyngoscopy and tympanometric evaluation.

### 2.3. Endoscopy

**Figure 1:**
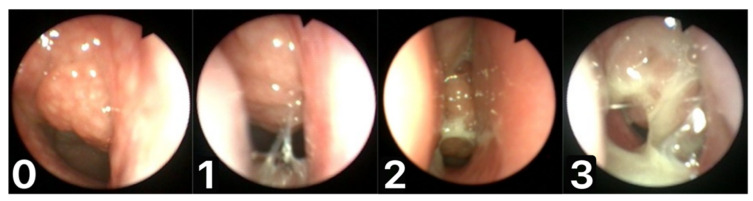
Mucus of Adenoid Scale by Nasopharyngoscopy Assessment (MASNA): ((**0**)—no mucus; (**1**)—residue of clear watery mucus; (**2**)—some amount of dense mucus; (**3**)—copious thick dense mucus).

Flexible fibreoptic rhinoscopy examinations were performed by an ENT children specialist (A.Z.) doctor with over 15 years of experience using the Karl Storz Germany, Tele Pack compact endoscopy system (18 kilopixels, 2.8-mm outer diameter, flexible nasopharyngoscope; Medit Inc., Winnipeg, MB, Canada). Based on the recorded video files, we used DaVinci Resolve 17 software (Blackmagic Design) to evaluate and calculate the percentages of obturation of the choanae (A/C ratio, adenoid-to-choana ratio in percentage) and analysed mucus coverage of the adenoids. The A/C ratio was assessed with an accuracy of up to 5% and then classified according to the Bolesławska scale. Mucous coverage of the adenoids was classified according to a previously devised and described scale (Figure 1) called the Mucus of Adenoid Scale by Nasopharyngoscopy Assessment (MASNA) [18]. This is a four-point scale describing the amount of mucus covering the adenoid: 0 = no mucus, 1 = residue of clear watery mucus, 2 = some amount of dense mucus, 3 = copious thick dense mucus. The adenoid size and mucus coverage recorded on the endoscopic system before and after treatment were blindly assessed by a second doctor (K.M.), and then the results were compared with those scored by first doctor (A.Z.). If there was a discrepancy in the assessment, the score was reassessed by a third ENT doctor (P.B.). We statistically considered the total number of patients depending on the amount of adenoid reduction, ingrowth and lack of change.

The change in adenoid size was considered as the percentage difference in the A/C ratio before and after steroid treatment. The degree of change in adenoid mucus on the MASNA scale was also assessed. A higher score of change in adenoid mucus scale represented an increase in mucus coverage, while a lower score of change indicated decreased mucus coverage.

### 2.4. Tympanometry

To evaluate effusion in the middle ear, we analysed the type of tympanograms based on the Jerger classification before and three to six months after treatment [19]. A shift from a Type B to a Type C/A tympanogram or from a Type C to a Type A tympanogram was considered an improvement. The persistence of the same type of tympanogram was considered as no change. A shift from a Type C to a Type B or from a Type A to a Type C/B was considered a deterioration.

### 2.5. Medical Treatment

After the first visit, patients received a 12-week course of conservative treatment with mometasone furoate nasal spray and saline irrigation, which are a standard pharmacological treatment for adenoid hypertrophy symptoms [11,14,20]. One hundred micrograms of the steroid were puffed in each nostril once daily before sleep. We recommended use of hypertonic saline misting sprays twice a day to each nostril, the second dose was administrated at least 15 min before steroid puffs.

We analysed the late changes in the adenoids and tympanometry after topical steroid treatment with a leeway period of three to six months without drug intake.

### 2.6. Statistical Analysis

We used descriptive statistics to summarize and describe variables for the study group. We summarized quantitative variables, such as age and adenoid size, based on their means ± standard deviation (SD) and medians using the 25th and 75th percentiles (Q25–Q75). For the categorical variables, including gender, mucus coverage according to the MASNA scale, adenoid size according to the Boleslavska scale and tympanograms, we used frequency counts and percentages. In order to determine the differences between the independent variables, statistical significance was estimated using the Chi-square method or Fisher’s exact test for categorical variables and a Student’s t-test for quantitative variables. To determine the impact of the steroid treatment and thermal seasons on adenoid size, adenoid mucilage coverage and tympanogram we used analysis for the dependent variables. Quantitative variables were compared with a Student’s *t*-test tests for paired samples. The McNemar–Bowker test was used for analysis of categorical variables.

To multivariate analysis of the associations between other factors determined during the first medical visit and intranasal corticosteroid response such as gender, sequence of examination, and age, adenoid size (C/A ratio), adenoid mucus coverage and tympanogram as category variables, logistic regression analysis was performed. Odds ratios (OR) and 95% confidence intervals (95% CIs) were also calculated for considered clinical variables in regression models.

Intranasal corticosteroids response we assessed in three separate analyses: by improvement in (1) adenoid size (C/A ratio), (2) adenoid mucus coverage and (3) tympanogram, as the binary clinical variables. Adenoid size (C/A ratio) improvement was defined as a decrease in value of the C/A ratio by at least 15% from baseline (assessed at first visit). Adenoid mucus coverage improvement was defined as achievement of the lower category of mucilage in MASNA scale from baseline. Tympanogram improvement was based on a three-categorical tympanogram variable: A, B or C and was defined as achievement of better category from baseline (detection of changes: from B to A, B to C and C to A). As these binary variables were created by comparing the results before and after therapy, in the regression models the relevant baseline variables were not included in the creation of individual models.

For all these tests, two-tailed *p*-values were used, and differences at the level of *p* < 0.05 were considered significant. All statistical analyses were performed with SPSS (Statistical Package for the Social Sciences version 26, Armonk, NY, USA) software.

### 2.7. Ethics

Ethical approval for this study was obtained from the ethics committee of Nicolaus Copernicus University (KB 581/2021).

## 3. Results

We analysed data from the sequential pediatric ENT examinations of 165 children in the age group of 3–6 years, (mean age 4.14 ± 0.97) before and three to six months after treatment with mometasone furoate, an intranasal topical steroid (Table 1). Of the children, 83 (50.30%) were girls and 82 (49.70%) were boys. The demographic and clinical findings of the patients are summarized in Table 1.

We analysed the impact of steroids on the change in the A/C ratio, adenoid mucus and tympanometry for each patient. The A/C ratio decreased in 53 (32.12%) children, remain unchanged in 62 (37.58%) and increased in 50 (30.30%) (Table 1). The mean A/C ratio before treatment was 65.73%, and three to six months after finishing the 12-week course of intranasal steroid treatment the ratio decreased to 65.52% (*p* = 0.743) (Table 2). There were no statistically significant differences found when comparing selected groups of the same grade of adenoid hypertrophy using the Bolesławska scale (*p* = 0.541). Only in one patient did adenoid size decrease three to six months after treatment to the first degree in the Bolesławska scale (Table 2). We did not observe any change in adenoid mucus based on the MASNA scale before and three to six months after the end of the steroid treatment (*p* = 0.894) (Table 2). Moreover, long-term observations of tympanograms before and three to six months after the end of the treatment did not show improvement in tympanometry (*p* = 0.428) (Table 2). There was no change on tympanometry results. From a total of 80 (48.48%) children, there was improvement in 32 (19.39%) children and deterioration in 53 (32.12%) (Table 1).

Comparing the efficacy of the steroid on adenoid mucous coverage and tympanometry depending on the season in which the intranasal steroid was administrated, we observed statistically significant differences in the changes in adenoid mucus coverage (*p* = 0.002) and in tympanograms (*p* = 0.000000505) (Table 3). A statistically significant impact of thermal season on adenoid mucus coverage was confirmed by the MASNA scale (*p* = 0.003) and tympanograms (*p* = 0.0000548), but there was no impact of season on adenoid size (*p* = 0.280) (Table 4).

As shown, sequence of examination associated with different thermal seasons affects the final therapeutic outcome, evaluated by analysing adenoid size (C/A ratio), adenoid mucus coverage and tympanogram before and at last 12 weeks after completing the treatment. Therefore, we subsequently analysed which factors besides thermal sequence of examination affect the assumed response rates using logistic regression analysis. For this analyses adenoid size (C/A ratio), adenoid mucus coverage and tympanogram was categorised as the binary clinical variables (improvement vs. no improvement). A detailed description of the adopted criteria for indicating improvement for these variables can be found in the in statistical analysis section.

According with assumed criteria, improvement for adenoid size (decrease C/A ratio by at least 15%) was detected in 53 (32.12%), for adenoid mucus coverage in 55 (33.33%) and for tympanogram in 53 children (32.12%).

Logistic regression analyses have shown that the assumed variables have no significant effect on reducing the adenoid size. We showed that, the process of achieving of the adenoid mucus coverage improvement is significantly influenced only by the sequence of examination. The obtained estimates indicate that patients from the winter→summer (W/S) group have more than 2.5 times greater chance of obtaining a favorable result compared to patients from the summer→winter (S/W) group (OR [odds ratio] = 2.86, 95% CI [95% confidence internal] = 1.38–5.91).

Beside sequence of examination, the gender, baseline adenoid size (C/A ratio) in Bolesławska scale and baseline adenoid mucus coverage (MASNA scale) were showed as significant covariates in the model assessing the achievement of tympanogram improvement, with the greatest importance for the sequence of examination. Resulting data showed that conducting therapy and analysing the results of treatment in the winter–summer regimen is about seven times more effective in improving hearing compared to patients treated in the reverse thermal regimen (OR = 7.08, 95% CI = 2.89–17.31) (Table 5).

## 4. Discussion

Our study based on 165 children with adenoid hypertrophy treated with mometasone furoate did not reveal any change in adenoid size and its mucus three to six months after finishing a 12-week course of intranasal steroid treatment (*p* = 0.541, *p* = 0.894 respectively). Additionally, there were no differences in middle ear effusion on tympanometry examination (*p* = 0.428).

These results are in contrast to those of the vast majority of studies, which have shown reductions in adenoid hypertrophy and related symptoms after the use of intranasal steroids [10,11,14,21,22,23,24,25,26,27,28]. One exception is the work of Anjali Lepcha, but her results showing no effect of beclomethasone on tonsils was based on an analysis of only 13 patients, and the results were not statistically significant (*p* < 0.060) [29]. None of these trials established the optimal duration of the treatment in children. Almost all of them evaluated adenoid size immediately following conservative treatment and not after a leeway period without topical steroid treatment. The clinical findings of the previously published papers are summarized in Table 6.

Only a few papers mentioned the long-term effects of adenoid hypertrophy treatment with intranasal steroids and only those study could be used to assess if steroid therapy could prevent the need for surgery. The longest follow-up period of 31 months was presented by Berlucchi [20]. In his study, he advised an increase in the duration of steroid treatment to 31 months and proposed a different schema of steroid therapy: two weeks of intranasal steroids taken every month, suspended during the summer. He claimed this could free children from adenoid symptoms and reduce adenoid size. The study group was relatively small, containing only 12 patients, and this treatment may not be acceptable because of the potential side effects. A larger study, with a group of 53 patients and a long follow-up time of up to two years, was presented by Criscuoli. However, despite a clear improvement in the health of 45% of the children as a result of steroid treatment, 70% of them needed to undergo adenoid surgery at the end of the study [11]. Jazi also reported clinical improvement in patients eight weeks after the end of treatment, but the study group was limited to 20 patients [28]. Arguably the most reliable study was presented by Bhargava [10], who analysed a large cohort (100 children) over a long follow-up period (six months) after the end of steroid therapy. The endoscopic evaluation results indicated a long-term reduction in the size of the adenoid as a result of treatment. Unfortunately, the group of children aged 2–5 years contained only 13 patients. Generally, studies analysing changes in the size of the pharyngeal tonsil in children treated with intranasal steroids have employed small samples, with only three including samples exceeding 100 children (Table 6). This makes proper inference challenging.

It seems that in order to achieve the beneficial effect of treatment and avoid surgery, steroids should be used for a longer time over a period of up to several years with possible periods of suspension when symptoms are reduced. This period may be summer, where we observed an improvement in adenoid mucus coverage and tympanometry results [18]. The scheme proposed by Berlucchi may warrant consideration as well [20]. However, research is required on a larger group of preschool children to determine the lowest effective dose as well as the side effects of long-term steroid use. Local adverse effects in children, such as nasal irritation, sneezing, epistaxis, a burning and dry sensation in the nose and septal perforation, are uncommon [30,31]. The use of mometasone fluorinate for one year in children aged six to nine years showed only minimally higher rates of epistaxis in the sample group compared to a placebo, and there has been no study focused on preschool children [32]. Moreover, parents should be informed not to administer intranasal topical steroids with topical decongestants to their children for longer than a few days because of tachyphylaxis and rebound congestion [30]. Intranasal steroids may influence children’s growth or bone metabolism. Such an effect was shown in a study analysing beclomethasone [33]. However, a study on a newer intranasal steroid (mometasone furoate) at a dose of 100 micrograms per day in 98 children three to nine years of age contradicted these findings [32]. One year of continuous steroid treatment did not retard the growth of the children [32]. However, McDonnell concluded that children using intranasal steroids on a regular basis should be checked for growth, following clinical growth charts (CDC) growth curves. This may be performed by the child’s pediatrician [30].

This study confirmed our previous performed study concerning on seasonality change of adenoid size and mucus coverage and change in tympanogram [18]. Moreover, it shows that sequence of steroid treatment and observation may influence on positive results. We obtained a 2.5 times greater chance of favorable results for mucus coverage in the MASNA scale if we began treatment in winter and assessed the results in summer. It shows that seasonality impact on obtained results should be always taken into account if impacts on other factors are assessed on the adenoid change in a long-time period.

Age group selection is particularly important. Combining a group of children 2–6 years of age with a group 7–12 years of age may affect the results. In the younger age group, ailments associated with the pharyngeal tonsil are usually more burdensome and significant. In schoolchildren, these ailments are less bothersome [34].

In our long-term study, we found no improvement in tympanometry results after 12-week treatment with topical steroids. This is consistent with the international consensus based on 12 studies of 945 patients, which showed no improvement in long-term OME clinical symptoms after intranasal steroid treatment. Hence, steroid treatments are not recommended due to their cost, possible side effects and lack of long-term efficacy [35]. Tympanostomy ventilation tube insertion with or without adenoidectomy is the only treatment to have been validated by the international scientific community for persistent OME with functional impartment of hearing between 25 and 40 dB or with damage of tympanic membrane [36,37].

Recently, new publications on adenoid hypertrophy and OSA and the impact of adenoidectomy on the improvement of behavioral symptoms in children indicate the adverse effect of long-term impaired nasal ventilation on the child’s cognitivist development [38]. Confirmed in our study was the absence of long-term effectiveness of steroid pharmacological treatment of the adenoid hypertrophy and OSA symptoms indicate the need to look for another effective method of pharmacological treatment or to decide more quickly on adequate surgery.

## 5. Conclusions

The results indicate that there was no effect of intranasal mometasone furoate on adenoid size, its mucus and OME three to six months after finishing a 12-week course of treatment. Topical steroids seem to have a temporary effect on adenoid size and its mucus, which decreases when they are not used. The durability of the long-term effects of newer intranasal steroids used for a moderate period on adenoid size should be analysed and compared with those of steroids used for a longer duration to establish an effective treatment for adenoid hypertrophy and to avoid adenoidectomy. Further studies are needed on the impact of multiple-year courses of topical steroids on adenoid size and its mucus coverage as well as side effects on preschool children. We are inclined to use the Berrlucchi scheme, which reduces the risk of complications with long-term topical steroid use. We were not able to confirm the beneficial effect of intranasal steroids on tympanometry results reported in other studies. In light of the performed study decision, adenoidectomy and tympanostomy should not be procrastinated. The seasonality impact on the adenoid mucus coverage and tympanometry should be always taken into account if the impact of other factors is assessed on the adenoid change in a long-time period.

## Figures and Tables

**Table 1:** Patients characteristics.

Characteristic		All Patients
*n*		165
Age at the first visit (years)	mean ± SD	4.14 ± 0.97
	median (Q25–Q75)	4.00 (3.00–5.00)
Gender	female	83 (50.30%)
	male	82 (49.70%)
Sequence of examination	Summer→winter (S/W)	80 (48.48%)
	winter→summer (W/S)	85 (51.52%)
Adenoid size (A/C ratio and (Bolesławska scale)—first visit	mean ± SD [%]	65.73 ± 12.57
	median (Q25–Q75) [%]	65.00 (55.00–75.00)
	II	93 (56.36%)
	III	72 (43.64%)
Adenoid size (A/C ratio and (Bolesławska scale)—second visit	mean ± SD [%]	65.52 ± 12.68
	median (Q25–Q75) [%]	65.00 (60.00–75.00)
	I	1 (0.61%)
	II	88 (53.33%)
	III	76 (46.06%)
Impact of steroid on A/C ratio change	mean ± SD [%]	−0.21 ± 8.29
	median (Q25–Q75) [%]	0.00 (−5.00–5.00)
	decrease	53 (32.12%)
	no change	62 (37.58%)
	increase	50 (30.30%)
Adenoid mucus coverage (MASNA scale)—first visit	0	29 (17.58%)
	1	59 (35.76%)
	2	46 (27.88%)
	3	31 (18.79%)
Adenoid mucus coverage (MASNA scale)—second visit	0	26 (15.76%)
	1	58 (35.15%)
	2	51 (30.91%)
	3	30 (18.18%)
Impact of steroid on adenoid mucus coverage change	decrease	55 (33.33%)
	no change	48 (29.09%)
	increase	62 (37.58%)
Tympanogram—first visit	AA	74 (44.85%)
	AB/BA	9 (5.45%)
	AC/CA	16 (9.70%)
	BB	36 (21.82%)
	BC/CB	15 (9.09%)
	CC	15 (9.09%)
	A	74 (44.85%)
	B	60 (36.36%)
	C	31 (18.79%)
Tympanogram—second visit	AA	87 (52.73%)
	AB/BA	3 (1.82%)
	AC/CA	14 (8.48%)
	BB	30 (18.18%)
	BC/CB	11 (6.67%)
	CC	20 (12.12%)
	A	87 (52.73%)
	B	44 (26.67%)
	C	34 (20.61%)
Impact of steroid on tympanogram change	improvement	32 (19.39%)
	no change	80 (48.48%)
	deterioration	53 (32.12%)

First visit—visit before start of treatment; second visit—visit over 3 months after end of treatment, A/C ratio—adenoid to choana ratio.

**Table 2:** Impact of the intranasal steroid on the adenoid size, adenoid mucus coverage and tympanograms.

Characteristic		Intranasal Steroid Treatment		*p* Value
		First Visit (Before Beginning of Treatment)	Second Visit (From >3 to <6 Months after End of Treatment)	
Adenoid size (A/C ratio and (Bolesławska scale)	mean ± SD (%)	65.73 ± 12.57	65.52 ± 12.68	0.743
	median (Q25–Q75) (%)	65.00 (55.00–75.00)	65.00 (60.00–75.00)	
	I	0 (0.00%)	1 (0.61%)	0.541
	II	93 (56.36%)	88 (53.33%)	
	III	72 (43.64%)	76 (46.06%)	
Adenoid mucus coverage (MASNA scale)	0	29 (17.58%)	26 (15.76%)	0.894
	1	59 (35.76%)	58 (35.15%)	
	2	46 (27.88%)	51 (30.91%)	
	3	31 (18.79%)	30 (18.18%)	
Tympanogram	AA	74 (44.85%)	87 (52.73%)	0.428
	AB/BA	9 (5.45%)	3 (1.82%)	
	AC/CA	16 (9.70%)	14 (8.48%)	
	BB	36 (21.82%)	30 (18.18%)	
	BC/CB	15 (9.09%)	11 (6.67%)	
	CC	15 (9.09%)	20 (12.12%)	
	A	74 (44.85%)	87 (52.73%)	0.126
	B	60 (36.36%)	44 (26.67%)	
	C	31 (18.79%)	34 (20.61%)	

**Table 3:** Impact sequence of examination on clinical and demographic variables.

Characteristic		The Sequence of Examination		*p* Value
		Summer→Winter (S/W)	Winter→Summer (W/S)	
*n*		80 (48.48%)	85 (51.52%)	
Age at the first visit (years)	mean ± SD	3.99 ± 0.92	4.28 ± 1.00	0.050
	median (Q25–Q75)	4.00 (3.00–5.00)	4.00 (4.00–5.00)	
Gender	female	36 (45.00%)	47 (55.29%)	0.186
	male	44 (55.00%)	38 (44.71%)	
Adenoid size (A/C ratio and (Bolesławska scale)—first visit	mean ± SD (%)	65.00 ± 11.72	66.41 ± 13.35	0.473
	median (Q25–Q75) (%)	65.00 (55.00–75.00)	65.00 (55.00–80.00)	
	II	47 (58.75%)	46 (54.12%)	0.549
	III	33 (41.25%)	39 (45.88%)	
Adenoid size (A/C ratio and (Bolesławska scale)—second visit	mean ± SD (%)	65.50 ± 13.54	65.53 ± 11.90	0.988
	median (Q25–Q75) (%)	65.00 (60.00–75.00)	65.00 (60.00–75.00)	
	I	1 (1.25%)	0 (0.00%)	0.695
	II	41 (51.25%)	47 (55.29%)	
	III	38 (47.50%)	38 (44.71%)	
Impact of steroid on A/C ratio change	mean ± SD (%)	0.50 ± 8.37	−0.88 ± 8.21	0.286
	median (Q25–Q75) (%)	0.00 (−5.00–5.00)	0.00 (−5.00–0.00)	
	decrease	23 (28.75%)	30 (35.29%)	0.268
	no change	28 (35.00%)	34 (40.00%)	
	increase	29 (36.25%)	21 (24.71%)	
Adenoid mucus coverage (MASNA scale)—first visit	0	18 (22.50%)	11 (12.94%)	**0.003**
	1	35 (43.75%)	24 (28.24%)	
	2	20 (25.00%)	26 (30.59%)	
	3	7 (8.75%)	24 (28.24%)	
Adenoid mucus coverage (MASNA scale)—second visit	0	9 (11.25%)	17 (20.00%)	0.308
	1	28 (35.00%)	30 (35.29%)	
	2	25 (31.25%)	26 (30.59%)	
	3	18 (22.50%)	12 (14.12%)	
Impact of steroid on adenoid mucus coverage change	decrease	16 (20.00%)	39 (45.88%)	**0.002**
	no change	26 (32.50%)	22 (25.88%)	
	increase	38 (47.50%)	24 (28.24%)	
Tympanogram—first visit	AA	47 (58.75%)	27 (31.76%)	**0.004**
	AB/BA	6 (7.50%)	3 (3.53%)	
	AC/CA	7 (8.75%)	9 (10.59%)	
	BB	10 (12.50%)	26 (30.59%)	
	BC/CB	5 (6.25%)	10 (11.76%)	
	CC	5 (6.25%)	10 (11.76%)	
	A	47 (58.75%)	27 (31.76%)	**0.002**
	B	21 (26.25%)	39 (45.88%)	
	C	12 (15.00%)	19 (22.35%)	
Tympanogram—second visit	AA	35 (43.75%)	52 (61.18%)	0.106
	AB/BA	1 (1.25%)	2 (2.35%)	
	AC/CA	7 (8.75%)	7 (8.24%)	
	BB	21 (26.25%)	9 (10.59%)	
	BC/CB	5 (6.25%)	6 (7.06%)	
	CC	11 (13.75%)	9 (10.59%)	
	A	35 (43.75%)	52 (61.18%)	0.062
	B	27 (33.75%)	17 (20.00%)	
	C	18 (22.50%)	16 (18.82%)	
Impact of steroid on tympanogram change	improvement	23 (28.75%)	9 (10.59%)	**0.000000505**
	no change	10 (12.50%)	43 (50.59%)	
	deterioration	47 (58.75%)	33 (38.82%)	

First visit—visit before start of treatment; second visit—visit over 3 months after end of treatment.

**Table 4:** Impact of the thermal season on the adenoid size, adenoid mucus coverage and tympanograms.

Characteristic		Thermal Season		*p* Value
		Winter	Summer	
Adenoid size (A/C ratio and (Bolesławska scale)	mean ± SD (%)	65.97 ± 13.41	65.27 ± 11.78	0.280
	median (Q25–Q75) (%)	65.00 (60.00–80.00)	65.00 (60.00–75.00)	
	I	1 (0.61%)	0 (0.00%)	0.307
	II	87 (52.73%)	94 (56.97%)	
	III	77 (46.67%)	71 (43.03%)	
Adenoid mucus coverage (MASNA scale)	0	20 (12.12%)	35 (21.21%)	**0.003**
	1	52 (31.52%)	65 (39.39%)	
	2	51 (30.91%)	46 (27.88%)	
	3	42 (25.45%)	19 (11.52%)	
Tympanogram	AA	62 (37.58%)	99 (60.00%)	**0.0000548**
	AB/BA	4 (2.42%)	8 (4.85%)	
	AC/CA	16 (9.70%)	14 (8.48%)	
	BB	47 (28.48%)	19 (11.52%)	
	BC/CB	15 (9.09%)	11 (6.67%)	
	CC	21 (12.73%)	14 (8.48%)	
	A	62 (37.58%)	99 (60.00%)	**0.00000819**
	B	66 (40.00%)	38 (23.03%)	
	C	37 (22.42%)	28 (16.97%)	

**Table 5:** Factors associated with the adenoid size (C/A ratio), adenoid mucus coverage and tympanogram in patients treated with intranasal corticosteroids (logistic regression models).

Characteristic	*p* Value	OR	95% CI
**C/A ratio improvement (≥15% C/A ratio decrease from baseline)**			
Gender, male	0.602	0.83	0.42–1.65
Age, per year	0.297	1.20	0.85–1.70
The sequence of examination, winter→summer (W/S)	0.808	0.92	0.45–1.87
Baseline adenoid mucus coverage (MASNA scale), per category	0.067	1.43	0.98–2.10
Baseline tympanogram, per category	0.127	1.38	0.91–2.07
**Adenoid mucus coverage improvement (achievement of better category in MASNA scale from baseline)**			
Gender, male	0.850	1.07	0.53–2.14
Age, per year	0.661	1.08	0.76–1.54
The sequence of examination, winter→summer (W/S)	**0.005**	**2.86**	**1.38–5.91**
Baseline adenoid size (Bolesławska scale), per category	0.323	1.43	0.70–2.94
Baseline tympanogram, per category	0.051	1.50	0.99–2.25
Tympanogram improvement (achievement of better category from baseline *)			
Gender, male	**0.009**	**0.33**	**0.14–0.76**
Age, per year	0.325	0.82	0.55–1.22
The sequence of examination, winter→summer (W/S)	**0.000018**	**7.08**	**2.89–17.31**
Baseline adenoid size (Bolesławska scale), per category	**0.011**	**2.87**	**1.27–6.48**
Baseline adenoid mucus coverage (MASNA scale), per category	**0.002**	**2.04**	**1.31–3.16**

Baseline values were collected from data obtained at the first pre-treatment visit. * Analysis based on three-categorical tympanogram variables: A, B and C; tympanogram improvement if detection of changes: from B to A, B to C and C to A.

**Table 6:** Review of similar studies. MF—mometasone fluroate, F—flunisolide, B—beclomethasone.

Author Year Country	Age	Number of Patients Treated with Steroids	Medication	Time of Treatment	Time of Final Results Counting	Main Results
Cengel 2005 Turkey [21]	3–15	122	MF	6 weeks	at the end of therapy	42.2% improvement of OME
Ciprandi 2007 Italy [22]	3–6	139	F	8 weeks	at the end of therapy	reduction of A/H index in 72% children
Demirhan 2010 Turkey [14]	4–16	25	MF	8 weeks	at the end of therapy	symptoms improvement, 76% of children do not need adenoidectomy
Mohebbi 2014 Iran [23]	2–11 (2–4 and 5–11)	51	MF	3 months	at the end of therapy	improvement
Gupta 2015 India [24]	4–12	55	MF	4 weeks	at the end of therapy	improvement
Monga 2020 India [25]	3–11	30	MF	8 weeks	at the end of therapy	improvement
Rezende 2015 Brazil [26]	4–8	55	MF	6 weeks	at the end of therapy	reduction of adenoid size
Hassanzadeh 2016 Iran [27]	4–12	20	MF	4 weeks	at the end of therapy	reduction of adenoid size
Lepcha 2002 India [29]	3–12	13	B	8 weeks	at the end of therapy	**no improvement**
Berlucchi 2008 Italy [20]	3–7	21	MF	1–3 months before surgery or 15–31 months (mean 23) (2 weeks every month, suspended during the summer)	before surgery (9 children) or at the end of the maintenance therapy (12 children)	regular continuity MF therapy may obtain successful results
Criscuoli 2003 Italy [11]	Mean 3, 8	53	B	26 weeks	24,52,100 weeks after treatment	relevant clinical improvement in 45% children but 70% children performed surgery
Jazi 2011 Iran [28]	2–10	20	MF	6 weeks	1 and 8 weeks after treatment	clinical improvement more significant than adenoid regression in nasofiberoscopy
Bhargava 2014 India [10]	2–12	100	MF	24 weeks	24 weeks after treatment	clinical improvement

## Data Availability

Data available on request due to restrictions eg privacy or ethical. The data presented in this study are available on request from the corresponding author. The data are not publicly available due to protection of personal data.
